# Tomografia por Emissão de Positrões na Identificação de Infecção Associada a Dispositivos Cardiovasculares Implantáveis

**DOI:** 10.36660/abc.20260030

**Published:** 2026-07-22

**Authors:** Raquel Montalvão, Margarida Matias, Marisa Trabulo

**Affiliations:** 1 Departamento de Cardiologia Hospital de Santa Cruz Unidade Local de Saúde de Lisboa Ocidental Carnaxide Portugal Departamento de Cardiologia, Hospital de Santa Cruz, Unidade Local de Saúde de Lisboa Ocidental, Carnaxide – Portugal

**Keywords:** Tomografia por Emissão de Pósitrons combinada à Tomografia Computadorizada, Desfibriladores Implantáveis

**Figura 1:**
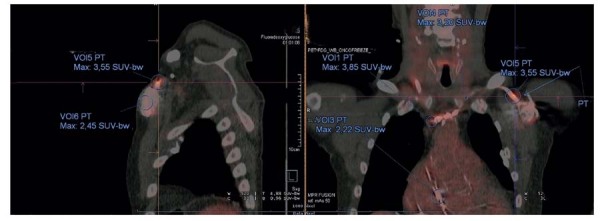
– Imagem PET/CT com fusão de 18F-FDG mostrando captação focal ao longo do eletrodo e gerador do marca-passo.

**Figura 2 f02:**
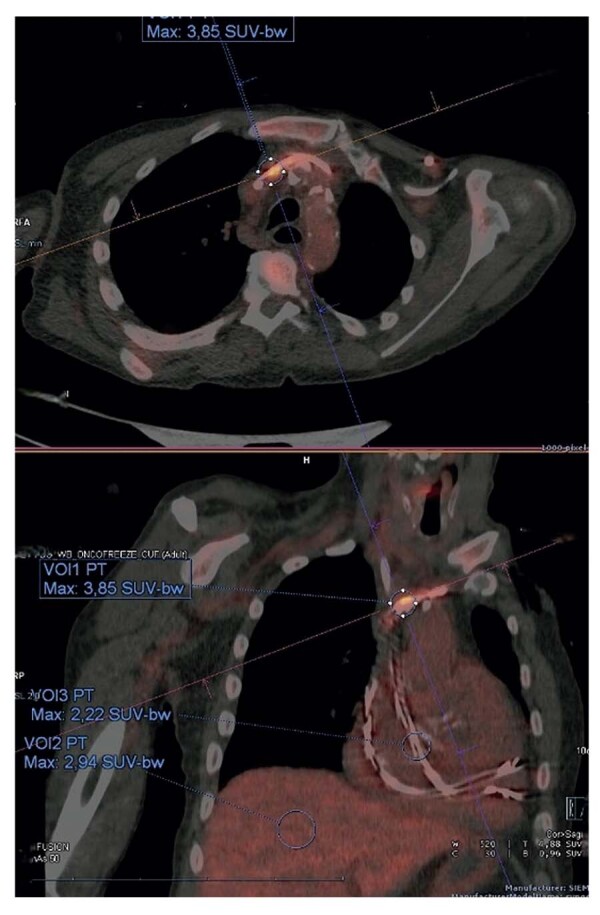
– Imagem PET/CT com fusão de 18F-FDG mostrando captação focal ao longo do eletrodo do marca-passo.

**Figura 3 f03:**
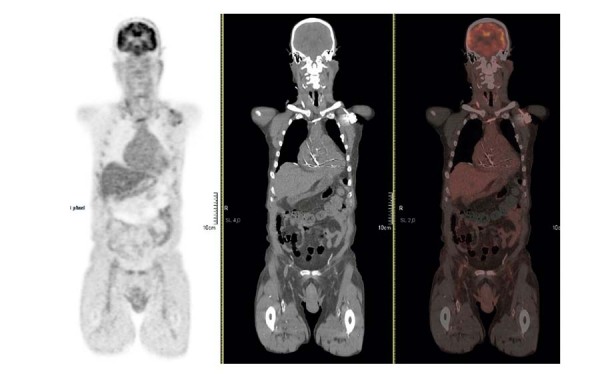
– Imagens coronais de corpo inteiro obtidas por PET/CT com 18F-FDG (PET, CT e fusão, da esquerda para a direita) mostram captação focal ao longo do eletrodo e gerador do marca-passo.

Um homem de 84 anos com hipertensão, dislipidemia e doença renal crônica, fibrilação atrial permanente e um marcapasso implantado devido à síndrome de bradicardia-taquicardia, com troca do gerador e dos eletrodos um ano antes, apresentou febre, mal-estar e fadiga por 15 dias. Ao exame físico, foi detectado sopro holossistólico. A pele sobrejacente à bolsa do marcapasso não apresentava sinais de inflamação. Os exames laboratoriais mostraram leucocitose com neutrofilia, proteína C-reativa de 9,5 mg/dL e procalcitonina de 1,3 ng/mL. O painel viral respiratório rápido foi negativo e a urinálise não apresentou alterações. Duas hemoculturas revelaram a presença de Staphylococcus aureus sensível à meticilina (MSSA). A tomografia por emissão de pósitrons/tomografia computadorizada (PET/CT) com 18F-fluorodesoxiglicose demonstrou aumento da captação ao longo do eletrodo e do gerador do marca-passo (Figuras 1-3 [Fig f02] [Fig f03]), confirmando a infecção do dispositivo eletrônico cardíaco implantável (DECI).Foi realizada a extração completa do sistema e as culturas confirmaram a presença de MSSA. Foram coletadas três hemoculturas de acompanhamento, duas antes da extração e uma após, todas negativas. O paciente recebeu cefazolina intravenosa por três semanas antes da extração e flucloxacilina intravenosa por três semanas após a extração, totalizando seis semanas de antibioticoterapia direcionada. O monitoramento revelou fibrilação atrial permanente sem pausas, e o reimplante do dispositivo não foi necessário.

O número crescente e a complexidade dos DECIs têm sido acompanhados por um aumento paralelo no risco de infecção.^1^ Em pacientes que apresentam doença sistêmica sem achados locais na bolsa do gerador, como neste caso, o diagnóstico de infecções de DECIs é desafiador.

A ecocardiografia continua sendo o método de imagem inicial; sua precisão diagnóstica é limitada. Tanto a abordagem transtorácica quanto a transesofágica podem não diferenciar ecogenicidades infecciosas de não infecciosas relacionadas a eletrodos, reduzindo a especificidade e levando a potenciais resultados falso-positivos. Além disso, a infecção por eletrodo pode ocorrer na ausência de massas visíveis no eletrodo, diminuindo ainda mais a sensibilidade.

A PET/CT é cada vez mais utilizada para detectar infecções de DECIs não identificadas por métodos de imagem convencionais. Ao integrar a atividade metabólica e inflamatória com dados anatômicos, a PET/CT melhora a precisão diagnóstica, atingindo uma especificidade relatada de aproximadamente 89-93% e sensibilidade de 83-98% em casos de infecção sistêmica com envolvimento do gerador e do eletrodo proximal.^1,2^ No entanto, ao avaliar infecções isoladas do eletrodo distal, a precisão diagnóstica é menor, com sensibilidade em torno de 39-65% e especificidade de 88-98%.^1,2^ A captação falso-positiva pode ocorrer após implante recente do dispositivo (<6 semanas),^3^ o que não se aplicava a este caso.

Documentos de consenso recentes da EHRA e da AHA agora enfatizam o papel da PET/CT em infecções duvidosas de DECIs. É particularmente útil na avaliação de pacientes com bacteremia persistente ou achados ecocardiográficos inconclusivos, onde essa modalidade pode detectar infecções subclínicas dos eletrodos e orientar a extração precoce do dispositivo, associada a melhores resultados clínicos.^3^

Apesar do seu uso crescente, ainda faltam ensaios clínicos randomizados e controlados para definir os regimes antibióticos ideais em infecções relacionadas a DECIs, o que reforça a necessidade de mais pesquisas nessa área.
