# Supplementary material for: Tomografia por Emissão de Positrões na Identificação de Infecção Associada a Dispositivos Cardiovasculares Implantáveis
Source: Arq Bras Cardiol. 2026 Jul 22;123(7):e20260030. [Article in Portuguese] doi: 10.36660/abc.20260030 (PMC13399541; doi:10.36660/abc.20260030)
Supplement: Translation

# Translation: Positron Emission Tomography Identification of Cardiovascular Implantable Electronic Device Infection

**DOI:** 10.36660/abc.20260030i

**Authors:** Raquel Montalvão, Margarida Matias, Marisa Trabulo

**Affiliations:** 1 Portugal Department of Cardiology, Hospital de Santa Cruz, Unidade Local de Saúde de Lisboa Ocidental, Carnaxide – Portugal

**Keywords:** Positron Emission Tomography Computed Tomography, Implantable Defibrillators

An 84-year-old man with hypertension, dyslipidemia, chronic kidney disease, permanent atrial fibrillation, and a pacemaker implanted for brady-tachy syndrome, with generator and lead replacement one year prior, presented with a 15-day fever, malaise, and fatigue. Physical examination revealed a holosystolic murmur. The skin overlying the pacemaker pocket showed no signs of inflammation. Laboratory testing showed leukocytosis with neutrophilia, C-reactive protein of 9.5 mg/dL, and procalcitonin of 1.3 ng/mL. The rapid respiratory viral panel was negative, and urinalysis was unremarkable. A pair of blood cultures yielded methicillin-susceptible *Staphylococcus aureus* (MSSA). Transthoracic and transesophageal echocardiography showed no valvular or lead involvement. 18F-fluorodeoxyglucose positron emission tomography /computed tomography (PET/CT) demonstrated increased uptake along the pacemaker lead and generator (Figure 1-3 [Fig f02001] [Fig f03001]), confirming cardiac implantable electronic device (CIED) infection. Complete system extraction was performed, and lead cultures confirmed MSSA. Three follow-up blood cultures were obtained, two before extraction and one after extraction, all of which were negative. He received 3 weeks of IV cefazolin pre-extraction and 3 weeks of IV flucloxacillin post-extraction, completing a total of 6 weeks of directed antibiotic therapy. Monitoring revealed permanent atrial fibrillation without pauses, and device reimplantation was not required.

**Figure 1:** – Fused 18F-FDG PET/CT image showing focal uptake along the pacemaker lead and generator.

**Figure 2 f02001:** – Fused 18F-FDG PET/CT image showing focal uptake along the pacemaker lead.

**Figure 3 f03001:** – Coronal whole-body views of 18F-FDG PET/CT imaging (PET, CT, and fused, from left to right) showing focal uptake along the pacemaker lead and generator.

The increasing number and complexity of CIED implantations have been accompanied by a parallel rise in infection risk.^1^ In patients presenting with systemic disease without local generator-pocket findings, as in this case, the diagnosis of CIED infections is challenging.

Echocardiography remains the initial imaging modality; its diagnostic accuracy is limited. Both transthoracic and transesophageal approaches may fail to differentiate infectious from noninfectious lead-related echodensities, reducing specificity and leading to potential false-positive results. In addition, lead infection can occur in the absence of visible lead masses, further lowering sensitivity.

PET/CT is increasingly used to uncover CIED infections not detected by conventional imaging methods. By integrating metabolic and inflammatory activity with anatomical data, PET/CT improves diagnostic accuracy, achieving a reported specificity of approximately 89-93% and sensitivity of 83-98% in cases of systemic infection with generator and proximal lead involvement.^1,2^ However, when assessing isolated distal lead infections, diagnostic accuracy is lower, with a sensitivity of around 39-65% and specificity of 88-98%.^1,2^False-positive uptake may occur after recent device implantation (<6 weeks),^3^ which was not applicable in this case.

Recent EHRA and AHA consensus documents now emphasize the role of PET/CT in equivocal CIED infections. It is particularly useful for the evaluation of patients with persistent bacteremia or inconclusive echocardiographic findings, where this modality can detect subclinical lead infections and guide early device extraction, associated with improved clinical outcomes.^3^

Despite its growing use, randomized controlled trials to define optimal antibiotic regimens in CIED infections remain lacking, underscoring the need for further research in this area.
